# Smaller subcortical volume in Parkinson patients with rapid eye movement sleep behavior disorder

**DOI:** 10.1007/s11682-018-9939-4

**Published:** 2018-08-28

**Authors:** Sanne Kamps, Odile A. van den Heuvel, Ysbrand D. van der Werf, Henk W. Berendse, Daniel Weintraub, Chris Vriend

**Affiliations:** 1 Department of Psychiatry, Amsterdam UMC, Amsterdam Neuroscience, Vrije Universiteit Amsterdam, Amsterdam, The Netherlands; 2 Department of Anatomy and Neurosciences, Amsterdam UMC, Amsterdam Neuroscience, Vrije Universiteit Amsterdam, Amsterdam, The Netherlands; 3 Department of Neurology, Amsterdam UMC, Amsterdam Neuroscience, Vrije Universiteit Amsterdam, Amsterdam, The Netherlands; 4 Departments of Psychiatry and Neurology, Perelman School of Medicine at the University of Pennsylvania, Philadelphia, PA USA; 5 Parkinson’s Disease and Mental Illness Research, Education and Clinical Centers (PADRECC and MIRECC), Philadelphia Veterans Affairs Medical Center, Philadelphia, PA USA; 6 Department of Anatomy and Neurosciences, Amsterdam UMC, VU University Medical Center, De Boelelaan 1108, P.O. Box 705, 1007 MB Amsterdam, The Netherlands

**Keywords:** Parkinson disease, REM sleep behavior disorder, Voxel-based morphometry, Structural covariance

## Abstract

Parkinson disease (PD) patients with rapid eye movement (REM) sleep behavior disorder (RBD) have worse motor symptoms and non-motor symptoms than patients without RBD. The aim of this study was to examine underlying differences in brain structure from a network perspective. Baseline data were obtained from Parkinson’s Progression Markers Initiative (PPMI) participants. We divided PD patients and healthy controls (HC) into RBD positive and RBD negative using a cutoff score of ≥5 on the RBD screening questionnaire. HC with probable RBD were excluded. We first carried out a region-of-interest analysis of structural MRIs using voxel-based morphometry to study volumetric differences for the putamen, thalamus and hippocampus in a cross-sectional design. Additionally, an exploratory whole-brain analysis was performed. To study group differences from a network perspective, we then performed a ‘seed-based’ analysis of structural covariance, using the bilateral dorsal-caudal putamen, mediodorsal thalamus and anterior hippocampus as seed regions. The volume of the right putamen was smaller in PD patients with RBD. RBD symptom severity correlated negatively with volume of the right putamen, left hippocampus and left thalamus. We did not find any differences in structural covariance between PD patients with and without RBD. Presence of RBD and severity of RBD symptoms in PD are associated with smaller volumes of the putamen, thalamus and hippocampus.

## Introduction

Rapid eye movement (REM) sleep behavior disorder (RBD) is a parasomnia characterized by vivid dreaming in combination with a loss of physiological muscle atonia, resulting in complex movements during REM sleep (Olson et al. 2000). Physiological muscle atonia during REM sleep is regulated by so-called ‘REM on’ and ‘REM off’ regions in the brainstem (Boeve 2010; Boeve et al. 2007). Dysfunction of these regions is thought to play a role in RBD pathophysiology (Boeve 2010; Boeve et al. 2007).

RBD is an early symptom of Parkinson’s disease (PD) and related disorders (Tekriwal et al. 2016), but not all PD patients display RBD. A recent meta-analysis showed a prevalence of 42.3% (Zhang et al. 2017). RBD in PD is associated with worse motor function and worse non-motor symptoms, such as constipation, olfactory dysfunction, excessive daytime sleepiness, cognitive impairment and a range of neuropsychiatric symptoms, including anxiety, depression, impulse control disorders and hallucinations (Arnaldi et al. 2016; Hu et al. 2015; Kim et al. 2014; Mahajan et al. 2014; Pacchetti et al. 2005; Rolinski et al. 2014).

Because of the differences in clinical profile between PD patients with RBD (PD RBD+) and without RBD (PD RBD-), several neuroimaging studies have explored potential underlying neurobiological differences. Idiopathic RBD (iRBD) is associated with smaller putamen volume (Ellmore et al. 2010, 2013). A voxel-based morphometry (VBM) study showed smaller thalamic volume in PD RBD+ (Salsone et al. 2014). A recent whole-brain deformation-based morphometry (DBM) study confirmed smaller putamen and thalamic volumes in PD RBD+, and additionally found a smaller volume of the pontomesencephalic tegmentum (PMT), medullary reticular formation (MRF), hypothalamus, amygdala and anterior cingulate cortex in PD RBD+ (Boucetta et al. 2016). Smaller hippocampal volume in PD RBD+ was reported in another VBM study (Lim et al. 2016).

These preliminary findings suggest that RBD in PD is associated with both (volumetric) alterations in the brainstem and subcortical and cortical degeneration. Boucetta et al. postulated that the observed structural differences not only reflect altered regulation of sleep-wake states and altered motor activity during REM sleep, but also play a role in the worse motor function and higher risk of neuropsychiatric symptoms associated with RBD (Boucetta et al. 2016).

The abovementioned studies found volumetric differences in isolated brain structures. However, the neurodegenerative process in PD may affect groups of interconnected brain areas (i.e., network-based neurodegeneration) rather than isolated brain areas independently, due to spreading of disease pathology to neighboring brain areas (Agosta et al. 2015). To test this assumption, the aim of this study was to further clarify neuroanatomical differences between PD patients with and without RBD in two ways; first, by studying volumetric differences in isolated brain structures, and second, by studying differences in connectivity from a brain network perspective. The secondary aim was to compare volumetric and connectivity parameters between the two patient groups and healthy controls (HC).

Based on the abovementioned literature we hypothesized that RBD in PD would be associated with degeneration of the putamen, thalamus and hippocampus. Therefore, we first carried out region-of-interest (ROI) analyses of these structures using VBM. Additionally, we performed an exploratory whole-brain VBM analysis to ensure we did not overlook significant group differences in regional volume.

To study group differences from a network perspective we used the relatively new technique of structural covariance (SC). SC can be used to study positive or negative covariance of gray-matter volume of one brain region with gray-matter volume of another brain region. Consistent patterns of covariance (when found across individuals) are considered to be indicative of connectivity between these brain regions. This is based on the theory that interconnected brain regions within a network not only show simultaneous brain activity, but also show correlations in gray-matter volume, perhaps due to joint neuroplastic or neurodegenerative processes (Mechelli et al. 2005). In line with this theory, structural covariance networks partially overlap with functional brain networks (He et al. 2007), and seed-based SC analyses of striatal and amygdalar sub-regions yield networks that are similar to functional networks (Montembeault et al. 2016; Soriano-Mas et al. 2013; Subira et al. 2016). Our aim was to determine whether there is a difference in SC between PD RBD+ and PD RBD-.

Within the abovementioned ROI’s (i.e., the bilateral thalamus, putamen and hippocampus) we selected smaller ‘seed regions’ involved in well-described functional networks that are believed to play a role in the pathophysiology of PD (i.e., limbic system, associative cortico-striatal-thalamo-cortical (CSTC)-circuit, and motor CSTC-circuit) (Nigro et al. 2016; Vriend et al. 2014). We hypothesized that PD RBD+ would show altered SC between each of the selected seeds regions and connected brain areas, suggesting altered connectivity.

## Materials and methods

### Participants

We made use of the open-access Parkinson’s Progression Markers Initiative (PPMI) database, a multicenter cohort study aimed at identifying biomarkers of PD progression. The aims and methodology of this study are published elsewhere (Marek et al. 2011) and are available at http://www.ppmi-info.org/study-design. The participants included in the PPMI database are de novo and treatment-naïve PD patients and age- and sex-matched HC. We only included data from the baseline visit. As polysomnography - the gold standard for RBD diagnosis - is not part of the PPMI study protocol, we divided PD subjects and HC into RBD+ and RBD- using the RBD screening questionnaire (RBDSQ). The RBDSQ is highly sensitive and reasonably specific for RBD (Stiasny-Kolster et al. 2007). In the present study, a score ≥ 5 (with specificity =90% and sensitivity =87% in PD) was defined as probable RBD (Stiasny-Kolster et al. 2015). HC with an RBDSQ score ≥ 5 were excluded. Patients with cognitive impairment (i.e. Montreal Cognitive Assessment (MoCA) score < 23) were also excluded (Nasreddine 2005).

### Analyses on demographic and clinical characteristics

We compared demographic and clinical characteristics between groups (HC, PD RBD- and PD RBD+). Neuropsychiatric and neuropsychological measures included the Epworth Sleepiness Scale (ESS) (Johns 1991), Geriatric Depression Scale (GDS-15) (Yesavage and Sheikh 1986), State-Trait Anxiety Inventory (STAI) (Spielberger 1983), Questionnaire for Impulsive-Compulsive Disorders in Parkinson’s disease (QUIP-S) (Weintraub et al. 2009) and MoCA (Nasreddine 2005). To compare motor symptom severity and disease stage, differences in the Movement Disorder Society Unified Parkinson Disease Rating Scale (MDS-UPDRS) III scores (Goetz et al. 2008) and Hoehn and Yahr (H&Y) stage (Hoehn and Yahr 1967) were tested in PD patients (PD RBD+ versus PD RBD-). Depending on the type of comparison (i.e., two-group or three-group comparison) and distribution of the variable, student t-tests, chi-square tests or one-way analyses of variance (ANOVA), followed by Hochberg’s post hoc comparisons, were performed in SPSS version 21 (SPSS, Chicago, IL, USA). Significance threshold was set at *p* < 0.05.

### Image acquisition

To minimize bias due to scanner differences, we selected the MRI scans acquired on a 3.0 Tesla MRI scanner from the same vendor (Siemens TrioTim 3.0 T and Siemens Verio 3.0 T). The PPMI board created an imaging manual to standardize data acquisition across the different study sites (details at http://www.ppmi-info.org/wp-content/uploads/2010/07/Imaging-Manual.pdf). Typical MRI parameters were: repetition time 5–11 ms; echo time 2–6 ms; slice thickness 1–1.5 mm; inter-slice gap 0 mm; voxel size 1x1x1.2 mm; matrix 256 x minimum 160.

### VBM preprocessing

We conducted the VBM analyses in SPM12 (Wellcome Trust Centre for Neuroimaging, London, UK) using the VBM8 toolbox (http://dbm.neuro.uni-jena.de/vbm.html) running in Matlab (MathWorks Inc., Natick, MA, U.S.A.). Quality of the scans was visually inspected by two independent raters, who were blinded to patient status, to exclude scans not suitable for further analysis (e.g., due to motion artifacts). Images were reoriented to the anterior/posterior commissural plane and segmented into grey matter, white matter and cerebrospinal fluid. A group-specific template was created using DARTEL (Diffeomorphic Anatomical Registration Through Exponentiated Lie Algebra; default settings). The segmented, modulated and normalised images were subsequently smoothed using a 10 mm FWHM Gaussian kernel. Quality checks on the segmented and normalised images, such as segmentation errors, were performed through visual inspection. Sample homogeneity was checked with tools implemented in the VBM8 toolbox.

### VBM analyses

We performed ROI analyses of the bilateral putamen, thalamus and hippocampus using VBM. To prevent false-negative results, we also performed exploratory whole-brain VBM analyses. To compare PD RBD+ and PD RBD-, we performed a two-group comparison using a two-sample t-test. To study correlations between regional volume and RBDSQ score, we performed multiple regression analyses comprising the entire group of PD patients. To compare PD RBD+ and PD RBD- patients to HC, we performed three-group analyses (PD RBD+ versus PD RBD- versus HC) through ANOVA models.

Age and sex were included in all statistical models as nuisance covariates and significance threshold was set at *p* < 0.05, after correction for multiple testing using family-wise error (FWE). Only contiguous clusters with >50 voxels were considered significant.

### Seed selection for structural covariance analyses

Although several methods are appropriate for assessment of SC (Evans 2013), currently the ‘seed-based’ approach is the most widely used. We selected our seed regions within the a priori selected ROI’s (i.e. the bilateral hippocampus, thalamus and putamen).

**Fig. 1:**
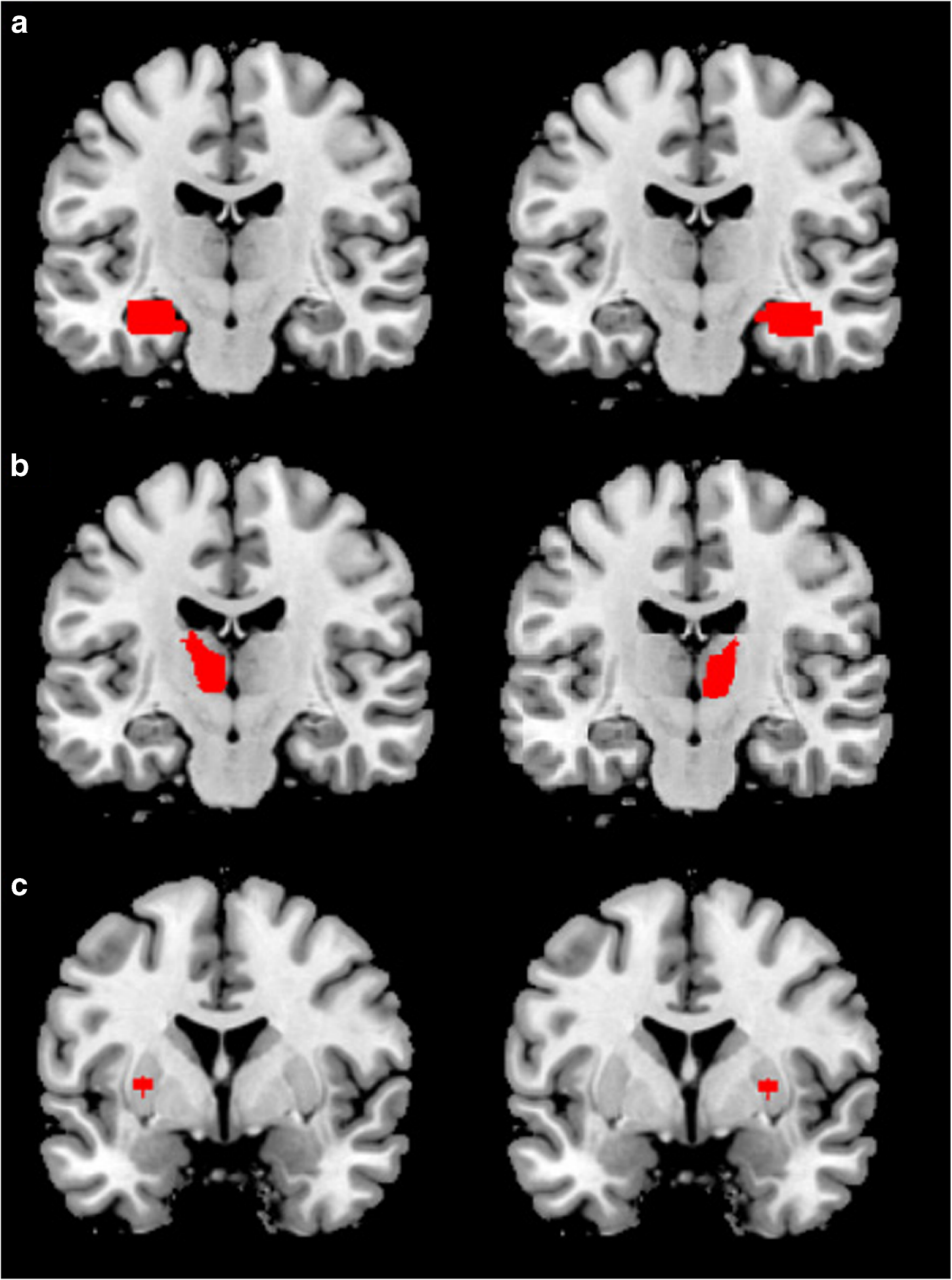
Selected seeds within bilateral hippocampus (**a**), thalamus (**b**) and putamen (**c**) for SC analyses

Within the hippocampus we selected the anterior hippocampus (AH) as seed region, because of its involvement in the limbic system, which is implicated in PD (Nigro et al. 2016). The definition of the AH was derived from literature (Persson et al. 2014). We modified the hippocampal region implemented in the Automated Anatomical Labelling (AAL) atlas, using the Wake-Forest University PickAtlas tool 3.0 (Fig. 1a).

Within the thalamus we selected the mediodorsal thalamus (MDT) as the seed region, because of its involvement in the associative CSTC-circuit, which is affected in PD (Vriend et al. 2014). The MDT was derived from the fsl-oxford-thalamic-connectivity-atlas (Behrens et al. 2003) (Fig. 1b).

Within the bilateral putamen we selected a 3.5 mm seed within the dorsal-caudal putamen (DCP), as previously described (Soriano-Mas et al. 2013). The DCP was selected because of its involvement in the motor CSTC-circuit, which is disturbed in PD and is correlated with motor symptom severity (Vriend et al. 2014). The MarsBar ROI toolbox was used to create a 3.5 mm sphere around MNI coordinates: x = ±28, y = 1, z = 3 (Fig. 1c). We extracted the grey volume of all six seeds for the SC analyses.

### Structural covariance analyses

Consistent with the VBM analyses, we primarily compared differences in SC between PD RBD+ and PD RBD- patients in a two-group comparison using two-sample t-tests. Additionally, multiple regression analyses were used to test for correlations with RBDSQ score in the entire sample of PD patients. Secondary three-group analyses (PD RBD+ versus PD RBD- versus HC) were performed through ANOVA models.

We built six separate SPM models, one for each seed region. All variables were orthogonalized using an iterative Gram-Schmidt method (Soriano-Mas et al. 2013). The models contained (in fixed order), the gray matter (GM) volume of the seed region, age and sex. The latter two were included as nuisance covariates. For the regression analyses, the orthogonalized RBDSQ score and the interaction between the orthogonalized RBDSQ scores and the seed volume (RBDSQ*volume) were additionally added to the model, consistent with Valk et al. (2016). We investigated both positive and negative correlations between the RBDSQ score and SC. Positive correlations would represent stronger SC between the seed region and other brain areas with increasing severity of RBD symptoms, while negative correlations would represent weaker SC between these regions. Statistical threshold was set at *p* < 0.05, FWE corrected.

## Results

### Demographic and clinical characteristics

**Fig. 2:**
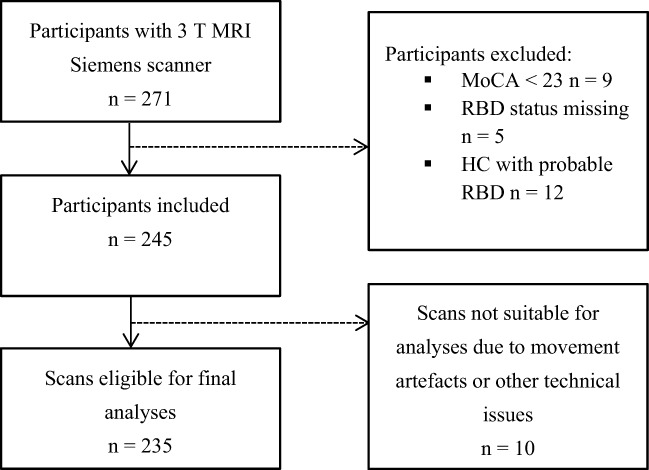
Flow chart depicting inclusion and exclusion of subjects

In total, 271 participants with scans acquired on a 3.0 Tesla MRI Siemens scanner were available, of which 26 met our exclusion criteria (see Fig.2). Because of movement artifacts or other technical issues 10 additional scans had to be excluded. A total of 235 subjects (40 PD RBD+, 127 PD RBD-, 68 HC) was eligible for final analyses.

Demographic and clinical data are listed in Table 1. Disease duration, H&Y stage and UPDRS III score did not significantly differ between PD RBD+ and PD RBD-. We did not find an effect of patient group (PD RBD+ versus PD RBD- versus HC) on sex, age, educational level, ESS, MoCA, GDS-15 or QUIP-S. A significant effect of groups was found on STAI score (*p* < 0.001). Post-hoc correlation analysis of RBDSQ and STAI score showed a significant (although weak) positive correlation between the two variables (*r* = 0.229; *p* = 0.003). GDS-15 score did significantly differ between HC and PD RBD+, but not between PD RBD+ and PD RBD-.

**Table 1:** Baseline demographic and clinical characteristics of participants

Variables	PD RBD+ (*n* = 40)	PD RBD- (*n* = 127)	HC (*n* = 68)	Test value (*p*-value)
Age (years)	61.10 (10.72)	61.08 (9.32)	59.41 (10.76)	F (2, 232) = 0.676 (0.509)^1^
Sex (male:female)	30:10	77:50	42:26	χ^2^ = 2.739 (0.107)^2^
Education (years)	15.60 (2.64)	15.45 (2.81)	16.18 (2.76)	F (2, 232) = 1.56 (0.21)^1^
Disease duration (months)	6.22 (7.69)	5.56 (6.92)	–	t (165) = 0.264 (0.608)^3^
Scanner type (TrioTrim:Verio)	39:1	117:10	63:5	χ^2^ = 1.410 (0.320)^2^
UPDRS III score	23.03 (9.84)	20.29 (8.93)	–	t (165) = 1.34 (0.101)^3^
H&Y stage (1:2:3)	16:24:0	55:70:2	–	χ^2^ = 0.837 (0.658)^2^
RBDSQ score	7.93 (1.49)	2.84 (1.35)	2.07 (1.53)	F (2, 232) = 238.83 (< 0.001)^1^* Post-hoc *p*-values (1) < 0.001; (2) 0.001; (3) < 0.001
ESS score	6.35 (3.87)	5.24 (3.10)	5.58 (3.58)	F (2, 230) = 1.653 (0.194)^1^
MoCA score	27.62 (1.68)	27.81 (1.77)	28.19 (1.19)	F (2, 232) = 1.897 (0.152)^1^
STAI score	70.65 (20.49)	63.01 (17.27)	56.10 (14.22)	F (2, 232) = 9.39 (< 0.001)^1^* Post-hoc p-values (1) 0.042; (2) 0.023; (3) <0.001
GDS-15 score	3.00 (2.92)	2.11 (2.35)	1.59 (2.99)	F (2, 230) = 3.57 (0.03)^1^** Post-hoc p-values (1) 0.186; (2) 0.470; (3) 0.024
Any impulse-control disorder (yes:no)	7:33	12:115	7:61	χ^2^ = 2.029 (0.168)^2^
Punding and / or hobbyism (yes:no)	6:34	12:115	10:58	χ^2^ = 0.437 (0.591)^2^

**I** Mean (standard deviations) are presented and statistically compared. The following statistical tests were used: ^1^ ANOVA; ^2^Pearson’s χ^2^ test; ^3^independent t-test

**II** When ANOVA showed significant between-group differences we performed a Hochberg’s GT2 post-hoc analysis to clarify which groups were significantly different: *significant effect of group; **although HC and PD patients show significant differences, no difference was found between PD RBD+ and PD RBD-. Post-hoc p-values represented: (1) between PD RBD- and PD RBD+, (2) between HC and PD RBD-, (3) between HC and PD RBD+

**III** Abbreviations: UPDRSIII: Unified Parkinson’s Disease Rating Scale part III; RBDSQ: REM sleep Behavior Disorder Screening Questionnaire; ESS: Epworth Sleepiness Scale; STAI: State-Trait Anxiety Inventory; GDS-15: Geriatric Depression Scale-15

### ROI analyses

In the two-group comparison, PD RBD+ patients showed smaller right putamen volume compared to PD RBD- (MNI coordinates: x = 30, y = −3, z = 12) (Table 2). To ensure that this was not explained by the difference in STAI score, we performed a post-hoc analysis in which we included STAI score in the statistical model as a nuisance covariate. This correction did not alter the observed between-group difference (data not shown). In the two-group comparison we did not find group differences for thalamus or hippocampus.

**Table 2:** Overview of VBM findings (ROI analyses)

Area	x	y	z	Cluster size	Cluster *p*-value (FWE-corrected)
PD RBD- > PD RBD+					
Putamen right	30	−3	12	305	0.031
Negative correlation with RBDSQ score					
Putamen right	30	−3	12	245	0.031
Hippocampus left	−14	−6	−14	69	0.030
	−16	−21	−18	51	0.033
Thalamus left	−15	−24	−3	51	0.036

Abbreviations: PD RBD+: Parkinson Disease patients with REM sleep Behavior Disorder; PD RBD-: Parkinson Disease patients without REM sleep Behavior Disorder; RBDSQ: REM sleep Behavior Disorder Screening Questionnaire; FWE: Family Wise Error

In the 3-group comparison, we found a significantly smaller right putamen volume in PD RBD+ patients compared to HC (MNI coordinates: x = 30, y = −9, z = −9, ke = 1449, pFWE = 0.009). There was no difference between PD RBD- and HC. Post-hoc exclusion of healthy controls with impulse control behaviors did not result in a different outcome.

**Fig. 3:**
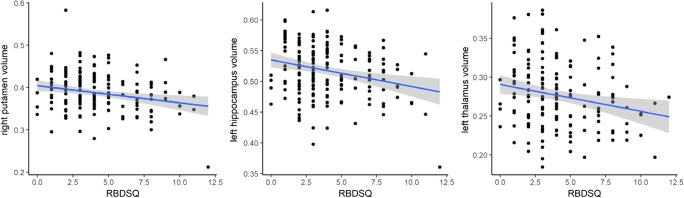
Scatterplots showing negative correlations between RBDSQ and right putamen, left hippocampus and left thalamus. Grey shade represents the 95% confidence interval. Abbreviations: RBDSQ: REM-sleep Behavior Disorder Screening Questionnaire

In the regression analysis (including all PD patients), RBDSQ score correlated negatively with volume of the right putamen (MNI coordinates: x = 30, y = −3, z = 12), left hippocampus (MNI coordinates: x = −14, y = −6, z = −14 and x = −16, y = −21, z = −18), and left thalamus (MNI coordinates: x = −15, y = 24, z = −3) (Table 2 and Fig. 3).

These results remained significant after correction for STAI score (right putamen (MNI coordinates: x = 27, y = −3, z = −9, ke = 2519, pFWE = 0.005); left hippocampus (MNI coordinates: x = −20, y = −8, z = −12, ke = 1313, pFWE = 0.001 and x = −14, y = −36, z = 3, ke = 158, pFWE = 0.021); left thalamus (MNI coordinates: x = −12, y = −12, z = −3, ke = 2398, pFWE = 0.001).

### Whole-brain analyses

The exploratory whole-brain analyses did not show any significant volumetric differences between PD RBD+ and PD RBD-, nor a correlation with RBDSQ score (after correction for multiple testing).

After post-hoc exclusion of healthy controls with impulse control behaviors, we observed a significant difference in volume between HC and PD-RBD+, located in the middle left temporal pole (MNI coordinates x = −33, y = 15, Z = −32, ke = 136, pFWE = 0.012), but no difference between PD RBD+ and PD RBD-.

### Structural covariance analyses

We did not find any differences in structural covariance between PD RBD+ and PD RBD-.

## Discussion

The aim of this study was to explore structural differences between PD patients with and without RBD and compare both patient groups to controls. We found that PD RBD+ patients show a smaller right putamen volume. Furthermore, we demonstrated a negative correlation between RBD symptom severity and right putamen, left thalamic and left hippocampal volume. For the first time, we also studied differences between PD RBD+ and PD RBD- from a network perspective. There were, however, no differences in structural covariance PD RBD+ and PD RBD- patients.

Smaller putamen volume was previously observed in iRBD (Ellmore et al. 2010, 2013), and Boucetta et al. recently found a smaller putamen volume in PD RBD+ as well (Boucetta et al. 2016). We replicated this finding. As was postulated by Boucetta et al., smaller putamen volume in PD RBD+ may reflect more pronounced degeneration of dopaminergic nigro-striatal pathways (Moustafa et al. 2017). Alterations in the putamen may in turn underlie the more severe motor symptoms associated with RBD in PD (Kim et al. 2014; Rolinski et al. 2014). Indeed, in our sample we found a significant negative correlation between putamen volume and motor symptom severity (data not shown). Interestingly, we did not find a significant difference in left putamen volume, which is in contrast with previous findings. This was also not evident when we tested at a less stringent threshold (*p*-value 0.001, uncorrected; data not shown). It is unclear whether our findings reflect actual asymmetry or have a different explanation.

The negative correlation between RBD symptoms severity and thalamic volume is in line with previous structural imaging studies of PD patients (Boucetta et al. 2016; Salsone et al. 2014). The thalamus is known to function as a ‘relay station’, between lower brain structures and the cortex, regulating various important brain functions, including sleep (Moustafa et al. 2017). The thalamus is innervated by cholinergic neurons originating in the pedunculo-pontine nucleus (PPN) and the dorsolateral tegmental nucleus (DLN) in the brain stem, nuclei that are implicated in RBD pathophysiology (Pepeu and Grazia Giovannini 2017). Interestingly, a positron emission tomography (PET) study in PD patients showed that RBD is associated with decreased thalamic cholinergic innervation by these nuclei (Pepeu and Grazia Giovannini 2017). Moreover, the presence of RBD predicts thalamic cholinergic deficits in PD (Muller et al. 2015). One may postulate that (more pronounced) cholinergic denervation of the thalamus in PD RBD+ is associated with loss of thalamic volume. Furthermore, a post-mortem study found more pronounced Lewy body pathology in the thalamus of PD patients with sleep disorders, including RBD, compared to PD patients without sleep disorders (Kalaitzakis et al. 2013). Extensive accumulation of Lewy bodies is associated with neurodegeneration and might in turn be associated with loss of volume. This might alternatively explain the smaller thalamic volume in PD RBD+.

The negative correlation between RBD symptom severity and hippocampal volume in PD patients is in line with preceding VBM studies (Kim et al. 2016; Lim et al. 2016). In contrast, Scherfler et al. showed a higher density of the bilateral hippocampus in iRBD patients, suggesting neuronal reorganization (Scherfler et al. 2011). It was shown previously that abnormal hippocampal perfusion in iRBD can predict conversion to PD (Dang-Vu et al. 2012). Although hippocampal alterations appear to be associated with PD-related RBD, it remains to be clarified whether these findings should be considered a primary mechanism or a secondary effect of the disorder.

In contrast with the ROI analyses, the whole-brain analyses showed no between-group differences, possibly due to our stringent correction for multiple testing. Nevertheless, this finding is in contrast with a recent PPMI whole-brain DBM-study, showing differences in PMT, MRF, hypothalamus, thalamus, putamen, amygdala and anterior cingulate cortex (Boucetta et al. 2016). This discrepancy might be the result of differences in sample size or difference in technique (DBM versus VBM). Also, brainstem areas were not represented in our GM mask.

Our study has several limitations. First, RBD diagnosis was not confirmed through polysomnography, the gold standard for a diagnosis of RBD. The RBDSQ that was used in the current study has a sensitivity of 0.96 and specificity of 0.56 for the diagnosis of RBD when a cut-off score of five points is used (Stiasny-Kolster et al. 2007). The use of the RBDSQ has been recommended by the EFNS/MDS-ES/ENS scientist panel (Berardelli et al. 2013). In spite of that, the lack of polysomnography could have affected our results. We could have overestimated PD patients with RBD and underestimated PD patients without RBD, which means that the PD RBD+ group could contain false positive patients.

Second, PPMI is a multicenter cohort study, and each study site used its own scanner. To minimize bias due to scanner differences, only MRI scans acquired on a 3.0 Tesla MRI scanner from the same vendor (Siemens) were selected. However, a bias introduced by inter-scanner variability remains a possibility.

Lastly, as we used a cross-sectional design, no conclusions with regard to causality can be drawn. It therefore remains unclear whether our findings indicate selective brain degeneration in PD causing RBD-associated symptoms, or that selective brain degeneration is a consequence of RBD. Longitudinal data, exploring structural differences between PD RBD+ and PD RBD- over time, will further increase our understanding of PD-related RBD in particular, and PD pathophysiology in general.

## Conclusion

Presence of RBD and severity of RBD symptoms in PD are associated with smaller volumes of the putamen, thalamus and hippocampus.
